# Orosomucoid 2 (ORM2) in type 2 diabetes and coronary artery disease: a potential link between insulin resistance and vascular inflammation

**DOI:** 10.3389/fcvm.2026.1859065

**Published:** 2026-06-25

**Authors:** Xia Sun, Xiang Tang, Guoyue Yuan

**Affiliations:** 1 Department of Cardiology, Affiliated Hospital of Jiangsu University, Zhenjiang, Jiangsu, China; 2 Department of Endocrinology and Metabolism, Affiliated Hospital of Jiangsu University, Zhenjiang, Jiangsu, China

**Keywords:** alpha-1-acid glycoprotein, atherosclerosis, coronary artery disease, hepatokine, ORM2, orosomucoid 2, type 2 diabetes mellitus, vascular inflammation

## Abstract

Type 2 diabetes mellitus (T2DM) and coronary artery disease (CAD) are closely linked cardiometabolic disorders that share common inflammatory, metabolic, and vascular mechanisms. However, the molecular mediators underlying their interconnected pathophysiology remain incompletely understood. Orosomucoid 2 (ORM2), a hepatocyte-derived acute-phase glycoprotein, has emerged as a potential mediator at the interface of metabolic and vascular dysfunction. Experimental and clinical evidence suggests that ORM2 plays important roles in hepatic lipid metabolism, insulin sensitivity, and immune regulation. Reduced ORM2 expression has been reported in obesity and insulin-resistant states, while circulating orosomucoid levels have been associated with diabetic nephropathy, microalbuminuria, and long-term risk of myocardial infarction. Mechanistically, ORM2 suppresses hepatic *de novo* lipogenesis through AMP-activated protein kinase signaling, improves glucose homeostasis by modulating interferon-*γ*/STAT1 signaling in adipose tissue, and regulates liver macrophage polarization via an inositol 1,4,5-trisphosphate receptor type 2-dependent calcium pathway. Preclinical studies further demonstrate that recombinant ORM2 attenuates atherosclerosis, hepatic steatosis, and steatohepatitis without detrimental metabolic effects, supporting its potential therapeutic relevance. Notably, ORM2 is regulated by pro-inflammatory cytokines, including interleukin-1β, interleukin-6, and tumor necrosis factor-α, which are central to the pathogenesis of both T2DM and CAD. Collectively, these findings position ORM2 as a promising integrative biomarker and therapeutic target within the adipose–liver–vascular axis. Further prospective clinical studies, Mendelian randomization analyses, and tissue-specific experimental models are needed to clarify its causal role in the shared pathophysiology of T2DM and CAD.

## 1 Introduction

Type 2 diabetes mellitus (T2DM) and coronary artery disease (CAD) together constitute a leading source of global morbidity and premature mortality. Cardiovascular disease (CVD) remains the foremost global cause of death (1). A large pooled analysis encompassing more than one million participants across Asian cohorts demonstrated that individuals with diabetes carried a 1.89-fold higher risk of all-cause mortality relative to non-diabetic individuals, with the risk of death specifically attributed to coronary heart disease elevated 2.57-fold (2). Atherosclerotic cardiovascular disease (ASCVD) is particularly prevalent and hazardous among persons with T2DM, reflecting a clinically significant bidirectional relationship in which metabolic dysfunction accelerates vascular pathology and vice versa. Despite extensive epidemiological characterization of this linkage, the molecular mediators that connect impaired metabolic control to vascular injury remain incompletely defined. Chronic inflammation and metabolic dysregulation have emerged as central drivers of atherosclerosis progression in this setting (1).

Atherosclerosis itself is a chronic inflammatory vascular disease initiated by endothelial activation and sustained by a cascade of molecular inflammatory responses that culminate in plaque formation and rupture (3, 4). A pivotal early event in endothelial dysfunction is activation of the nuclear factor-kappaB (NF-*κ*B) signaling axis, which orchestrates downstream expression of cytokines, chemokines, and adhesion molecules that amplify the vascular inflammatory response (5). C-reactive protein (CRP) serves as an established circulating marker of vascular inflammation in the coronary circulation (6). In metabolic disorders, chronically elevated interleukin-6 (IL-6) and tumor necrosis factor-alpha (TNF-α) perpetuate low-grade systemic and vascular inflammation, while glucotoxicity independently accelerates endothelial injury and promotes early atherosclerosis, as evidenced by the significant correlation between glycemic control and carotid intima-media thickness in diabetic subjects (7, 8).

Within this inflammation–metabolism interface, orosomucoid 2 (ORM2) has emerged as a candidate integrative mediator. ORM2 is a hepatocyte-derived acute-phase glycoprotein with pleiotropic roles in metabolic regulation (9, 10), and its characterization as a hepatokine underscores its capacity to mediate liver-to-adipose tissue crosstalk and systemic metabolic homeostasis (11). Hepatic and circulating ORM2 levels are markedly diminished in obese murine models and in patients with metabolic liver disease, and significant reductions in hepatic ORM2 expression have been documented in insulin-resistant states (10, 12). Mechanistically, ORM2 suppresses hepatic *de novo* lipogenesis through AMP-activated protein kinase (AMPK)-dependent signaling and enhances insulin sensitivity by inhibiting interferon-gamma (IFN-*γ*)/STAT1 signaling in adipose tissue (11, 12). Pharmacological administration of recombinant ORM2 markedly ameliorated atherosclerosis in preclinical mouse models (12), and ORM2 further modulates innate immune responses through macrophage reprogramming via an inositol 1,4,5-trisphosphate receptor type 2 (IP3R2)-dependent calcium signaling pathway (13).

Despite these multifaceted functions spanning lipid metabolism, insulin signaling, and vascular biology, ORM2 remains insufficiently characterized as a mechanistic bridge between metabolic dysfunction and coronary vascular inflammation, representing a significant gap in cardiometabolic research. Table 1 summarizes the structural, genetic, and functional differences between ORM1 and ORM2 that justify treating ORM2 as a metabolically specialized isoform rather than a redundant paralog of ORM1. Accordingly, this review pursues four primary objectives: (1) to synthesize current knowledge of ORM2 molecular biology and functional repertoire; (2) to critically appraise the existing evidence linking ORM2 dysregulation to the pathophysiology of T2DM and CAD; (3) to evaluate the potential of ORM2 as a clinical biomarker and therapeutic target in cardiometabolic disease; and (4) to delineate existing knowledge gaps and propose directions for future investigation.

**Table 1:** Head-to-head structural, genetic, and functional comparison of ORM1 and ORM2.

Feature	ORM1 (AGP-1)	ORM2 (AGP-2)	References
Protein identity/sequence	201 aa mature protein; ∼21 aa differ between isoforms; ∼89% sequence identity	201 aa mature protein; highly conserved across mammals; differs mainly in surface residues shaping drug pocket	(14, 15)
N-glycosylation	Five N-glycosylation sites (Asn15, 38, 54, 75, 85); carries bi-, tri-, and tetra-antennary complex glycans with variable sialylation and fucosylation	Same five N-glycosylation sites but different site occupancy and glycoform microheterogeneity; slightly more tetra-antennary chains reported	(14, 16)
Dominant tissue of origin	Hepatocytes; classic acute phase protein produced in response to systemic inflammation	Hepatocytes plus adipose tissue, leukocytes, endothelium, intestine and brain; behaves as a hepatokine and adipokine	(14, 17)
Regulation by bile acids/FXR	Strongly up-regulated by nuclear bile acid receptor FXR in hepatocytes	Also up-regulated by FXR signaling; ORM cluster shares hepatic bile acid response element	(18, 19)
Induction by IL-6/TNF-alpha	Robust positive acute phase induction (up to 3 to 5 fold) by IL-6 and glucocorticoids; smaller response to TNF-alpha	Induced by the same cytokine set in hepatocytes; ORM2 additionally induced in adipocytes under metabolic stress	(14, 20)
Drug binding specificity	Primary binder of basic and lipophilic drugs (propranolol, disopyramide, lidocaine) through a single high affinity site	Shares binding pocket but shows distinct affinities for methadone, chlorpromazine and some steroids; explains inter-individual PK variability	(16)
Immunometabolic function	Modulates leukocyte activity; binds pathogens; endogenous ligand carrier in inflammation	Interacts with leptin receptor and CCR5; integrates inflammatory and metabolic signals; implicated in AMPK activation in the liver	(14, 17)
Knockout/depletion phenotype	Orm1 knockout mice show aggravated NAFLD, hepatic inflammation and insulin resistance on high fat diet	Orm2 suppression worsens ischemic injury and metabolic stress responses; Orm2 overexpression improves glucose tolerance	(21, 22)
Dominant disease association	Acute inflammation, rheumatoid arthritis, sepsis, cancer progression through tumor associated macrophages	Metabolic dysfunction, obesity, T2DM, coronary artery disease, heart failure risk stratification	(23, 24)

aa, amino acid; AGP, alpha-1-acid glycoprotein; AGP-1/AGP-2, alpha-1-acid glycoprotein isoform 1/2; AMPK, AMP-activated protein kinase; CCR5, C-C chemokine receptor type 5; FXR, farnesoid X receptor; IL-6, interleukin-6; NAFLD, non-alcoholic fatty liver disease; ORM1/ORM2, orosomucoid 1/orosomucoid 2; PK, pharmacokinetic; T2DM, type 2 diabetes mellitus; TNF-α, tumor necrosis factor-alpha.

## 2 Biology of orosomucoid 2 (ORM2)

### 2.1 Gene structure and chromosomal location

The ORM2 gene, together with ORM1, belongs to a lipocalin subfamily encoded within the q32–34 region of human chromosome 9. Both genes are located in close proximity within this chromosomal cluster, which also encodes several other lipocalins with reported immunological functions, including neutrophil gelatinase-associated lipocalin, complement factor gamma-subunit, tear prealbumin, and prostaglandin D synthase (25). The ORM gene family includes ORM1 and ORM2, both of which encode acute-phase proteins referred to collectively as alpha-1-acid glycoprotein (AGP) or orosomucoid. The AGP gene is subject to transcriptional regulation through a combination of glucocorticoid-responsive elements and cytokine-driven mediators, and its expression in hepatocytes is modulated by a network involving interleukin-1 beta (IL-1β), TNF-α, IL-6, and IL-6-related cytokines (26). Regulatory elements within the AGP promoter region coordinate the response to both inflammatory and metabolic signals, consistent with the protein's dual role as an acute-phase reactant and metabolic regulator.

### 2.2 Protein structure and glycosylation

ORM2 is a member of the AGP family, with a molecular weight of 41–43 kDa and an isoelectric point (pI) of 2.8–3.8. The protein backbone consists of a single polypeptide chain of 183 amino acids in humans, stabilized by two disulfide bridges. Carbohydrate moieties contribute approximately 45% of the total molecular weight and are attached as five to six highly sialylated, complex-type N-linked glycans (26).

This extensive N-glycosylation gives rise to the characteristic charge heterogeneity and microheterogeneity observed across ORM2 glycoforms. Structural variation in the N-glycans, including differences in antennae branching, terminal sialylation, and fucosylation, generates the diverse glycoform repertoire of the protein (27). Importantly, the glycosylation profile of AGP is not static, as it changes in inflammatory and disease conditions, and altered sialylation has been documented to influence the protein's ligand-binding behavior in a glycoform-dependent manner (16, 28). These glycan-dependent structural changes are therefore relevant to the protein's biological function rather than to its biochemical identity alone.

### 2.3 Tissue expression and regulation

AGP is synthesized predominantly in the liver, where increased hepatic production drives the rise in circulating concentrations observed in response to systemic tissue injury, inflammation, or infection a pattern consistent with its classification as a positive acute-phase protein. Transcriptional regulation of the AGP gene is mediated by glucocorticoids together with a pro-inflammatory cytokine network primarily involving IL-1β, TNF-α, and IL-6, which together coordinate the acute-phase induction of hepatic expression (26).

Although hepatocytes are the dominant source, extrahepatic expression of AGP has also been reported in humans, and ORM2 specifically functions as a hepatokine capable of mediating liver-to-adipose tissue communication (11). This hepatokine activity provides a direct route by which hepatically derived ORM2 can influence peripheral metabolic tissues and contribute to systemic metabolic homeostasis.

Murine genetic models further support a metabolic role for ORM2 *in vivo*. High hepatic ORM2 expression is associated with resistance to high-fat diet–induced obesity, whereas Orm2 knockout mice develop spontaneous obesity under a standard chow diet and show exacerbation of diet-induced steatohepatitis (9).

Taken together, these regulatory features cytokine-driven hepatic induction, hepatokine-mediated liver-to-adipose crosstalk, and the metabolic phenotypes observed in genetic murine models position ORM2 as a regulator situated at the intersection of inflammatory and metabolic signaling, consistent with its membership in the immunocalin subfamily of lipocalins implicated in anti-inflammatory defense (25).

### 2.4 Known biological functions

AGP also binds and transports a range of lipophilic drugs in a glycoform-dependent manner (16, 26, 27, 29), although these pharmacological functions fall outside the metabolic-vascular focus of the present review.

While the downstream signaling effects of ORM2, including IP3R2-dependent calcium mobilization, AMP-activated protein kinase activation, and STAT1 suppression, have been characterized in increasing detail, the upstream mechanism by which secreted ORM2 first engages its target cells remains incompletely defined. As a circulating hepatokine, ORM2 must engage a cell-surface receptor or undergo cellular uptake to initiate intracellular signaling, yet no membrane receptor or intracellular binding partner specific to ORM2 has been formally identified and validated in the metabolic disease context. Whether ORM2 signals through pattern recognition receptors, lipocalin-binding partners, or a novel uncharacterized receptor system has not yet been established (11–13). This represents a core unresolved question in the field, and resolution of the upstream signal-initiation step is necessary to move from a descriptive understanding of ORM2 downstream effects to a complete mechanistic model of its action in cardiometabolic disease.

## 3 ORM2 in diabetes mellitus

### 3.1 ORM2 expression in type 2 diabetes and obesity

Direct human ORM2-specific evidence in metabolic disease has recently emerged from the KADEM study, a cross-sectional analysis of 449 Kuwaiti adults in which plasma ORM2 was measured directly by ELISA. In this cohort, elevated circulating ORM2 was significantly correlated with greater hepatic steatosis, insulin resistance, triglycerides, alanine aminotransferase, and hip circumference (*P* < 0.001), and individuals with severe steatosis (CAP > 290 dB/m) showed markedly higher ORM2 concentrations (312.3 ng/mL) than those with normal CAP scores (210.4 ng/mL; *P* < 0.001). After multivariable adjustment, ORM2 emerged as an independent predictor of steatosis severity (adjusted OR = 1.005; 95% CI: 1.002–1.007), and a composite ROC model incorporating ORM2 alongside metabolic variables achieved an AUC of 0.864 for metabolic dysfunction-associated fatty liver disease (30). These data establish ORM2 as a translationally relevant biomarker of metabolic dysfunction in humans, independent of the broader AGP signal.

Experimental data support and extend these human observations. Hepatic and circulating ORM2 concentrations are reduced in obese murine models and in patients with metabolic liver disease (12), Orm2 knockout mice develop spontaneous obesity under a standard diet (9), and hepatic ORM2 expression is significantly reduced in a high-fat diet plus letrozole model of insulin resistance (10). The directionality of the human and animal signals elevated circulating ORM2 in established metabolic dysfunction versus reduced hepatic expression in genetic and dietary models likely reflects the dual identity of ORM2 as both an acute-phase reactant induced by inflammation and a hepatokine whose intrinsic hepatic production is suppressed in metabolic stress. This bidirectional behavior is examined in greater detail in subsequent sections.

In contrast, the older clinical literature in T2DM is dominated by measurements of total alpha-1-acid glycoprotein (AGP) rather than ORM2 specifically. Serum AGP is elevated in T2DM patients with microalbuminuria relative to those with normoalbuminuria, and AGP concentrations correlate positively with urinary albumin excretion rate (r = 0.67, *P* < 0.01) (31). Because conventional immunoassays do not distinguish ORM1 from ORM2, these AGP-based findings should be regarded as indirect supportive evidence consistent with but not equivalent to ORM2-specific data. Dedicated clinical studies measuring ORM2 separately from total AGP in diabetic versus non-diabetic cohorts remain limited.

### 3.2 ORM2 and insulin resistance

Mechanistic studies indicate that ORM2 modulates insulin sensitivity and glucose metabolism through several converging pathways. To clarify the relative strength of evidence, the following discussion is organized hierarchically, with the principal mechanisms presented first and supporting or model-specific observations described thereafter.

The principal hepatic mechanism of ORM2 involves the IP3R2/AMPK/SREBP-1c axis. The best-characterized intracellular pathway of ORM2 in metabolic regulation involves direct binding to IP3R2, activation of AMPK, and downstream inhibition of SREBP-1c mediated lipogenic gene expression, resulting in suppression of hepatic *de novo* lipogenesis. This axis is supported by independent demonstrations that recombinant ORM2 administration ameliorates hepatic steatosis, steatohepatitis, and atherosclerosis *in vivo*, positioning it as the principal hepatic mechanism through which ORM2 exerts metabolic benefit (12).

The principal adipose mechanism of ORM2 involves suppression of IFN-*γ*/STAT1 signaling. In adipose tissue, hepatic ORM2 overexpression enhances systemic insulin sensitivity by suppressing IFN-*γ*/STAT1 signaling in inguinal white adipose tissue, an effect that partially recapitulates the metabolic actions of bile acids and improves glucose homeostasis (11). This pathway provides the principal mechanistic basis for ORM2's adipose-mediated insulin sensitization.

A supporting loss-of-function pathway involves the ERK1/2/PPAR*γ*/CD36 axis. Orm2 deletion in mice activates the ERK1/2/PPAR*γ*/CD36 signaling axis, increasing fatty acid uptake in hepatocytes (9). This pathway has been demonstrated primarily under knockout conditions and is best regarded as a compensatory or supporting mechanism that becomes evident in the absence of ORM2 rather than as a direct effector of ORM2 signaling.

A further model-specific observation concerns UCP1 in adipose tissue. In an insulin-resistant polycystic ovary syndrome (PCOS) model, Orm2 knockout mice exhibited hepatocyte hypertrophy and reduced uncoupling protein 1 (UCP1) expression in white adipose tissue, while recombinant ORM2 supplementation restored hepatocyte morphology and UCP1 expression (10). These observations derive from a single experimental context and are presented as model-specific supporting evidence rather than as a generalized mechanism.

Collectively, these animal data indicate that ORM2 modulates hepatic and adipose glucose and lipid metabolism through an integrated set of pathways anchored by IP3R2/AMPK/SREBP-1c in liver and IFN-*γ*/STAT1 suppression in adipose tissue. Direct contributions of ORM2 to canonical insulin signaling components, including GLUT4 translocation and the Akt/PI3 K axis, have not been characterized in ORM2-specific studies and represent a defined gap in the current mechanistic literature.

### 3.3 ORM2 and pancreatic beta-cell function

Direct evidence on ORM2 and pancreatic beta-cell function is not yet available in the published literature. Given ORM2's role as a cytokine-regulated acute-phase protein (26) and its modulation of insulin sensitivity in peripheral tissues (9, 11), its potential effects on the islet microenvironment in glucolipotoxic states warrant dedicated investigation.

### 3.4 ORM2 in diabetic complications

The most consistently reported association between orosomucoid and diabetic complications involves the kidney. A proteomic study identified orosomucoid as a urinary protein upregulated more than eightfold in diabetic nephropathy (DN) patients compared with healthy controls. Urinary orosomucoid excretion rate (UOER) increased progressively across normoalbuminuria, microalbuminuria, and macroalbuminuria groups. Multivariate logistic regression identified elevated UOER as an independent risk factor for DN, with an odds ratio of 3.10 (*P* < 0.0001). UOER was also positively correlated with urinary albumin excretion rate (r = 0.83) and serum creatinine (r = 0.79) (32). A separate urinary proteomics study similarly identified alpha-1 acid glycoprotein among protein markers present in urine samples of microalbuminuria-positive T2DM patients. In a cross-sectional clinical study, serum AGP was independently associated with microalbuminuria in T2DM patients (odds ratio = 1.16, 95% CI: 1.08–1.24, *P* < 0.001) (31).

### 3.5 ORM2 in gestational diabetes and type 1 diabetes

Data on ORM2 in gestational diabetes mellitus and type 1 diabetes are sparse and derive predominantly from total AGP measurements rather than ORM2-specific assays (26, 31). Direct interrogation of ORM2 in these populations represents a defined gap warranting prospective investigation.

## 4 ORM2 in coronary artery disease

### 4.1 ORM2 levels in CAD patients

In a study of 55 patients undergoing cardiac surgery, plasma orosomucoid concentrations were significantly higher in those with established CAD compared with patients without CAD (594 ± 207 vs. 412 ± 119 μg/mL), and epicardial adipose tissue–released orosomucoid showed a parallel elevation in CAD patients, suggesting that local coronary adipose inflammation contributes to circulating orosomucoid in CAD (32). Because this study measured total orosomucoid by routine immunochemical assay, the relative contributions of ORM1 and ORM2 to the observed elevation cannot be determined from the published data.

In acute heart failure, circulating total orosomucoid concentrations were associated with worse post-discharge outcomes. A combined model (OROME) incorporating orosomucoid and the adipokine omentin achieved a C-index of 0.84 for predicting death or heart failure readmission, compared with 0.80 for NT-proBNP alone (30). These findings indicate prognostic relevance for circulating orosomucoid in acute cardiac events, although ORM2-specific data across the spectrum of CAD are not yet available.

### 4.2 ORM2 and atherosclerosis: mechanistic insights

The most direct ORM2-specific evidence relevant to atherosclerosis comes from preclinical studies. Administration of recombinant ORM2 protein or an ORM2-Fc fusion protein markedly reduced atherosclerosis in mouse models without adverse effects on body weight or food intake, and this anti-atherogenic effect occurred alongside improvements in hepatic steatosis and steatohepatitis, suggesting a coordinated metabolic and vascular benefit (12, 33). ORM2 also reprograms liver macrophages through an IP3R2 dependent calcium signaling pathway (13). This macrophage-modulating activity is mechanistically relevant to atherosclerosis, in which polarization between pro-inflammatory and anti-inflammatory macrophage phenotypes influences plaque development and stability (3).

Direct ORM2-specific data on endothelial activation, monocyte adhesion, foam cell formation, and smooth muscle cell migration in CAD have not been reported in the published literature. The vascular-compartment effects of ORM2 in atherosclerosis therefore rest principally on the *in vivo* phenotypic outcomes of recombinant ORM2 administration rather than on direct interrogation of arterial-wall cell biology, and this gap is one of the most pressing in the field.

### 4.3 ORM2 and vascular inflammation

Atherosclerosis is initiated by endothelial activation, followed by NF-*κ*B-driven expression of cytokines, chemokines, and adhesion molecules within the arterial wall (3). At the family level, ORM2 and the broader AGP family are classified within the immunocalin lipocalin subfamily, which exerts a regulatory, dampening influence on the inflammatory cascade (25), and sialylation and N-glycan branching alter the protein's interactions with immune receptors (27). ORM2-specifically, suppression of IFN-*γ*/STAT1 signaling has been demonstrated in adipose tissue (11). Whether ORM2 modulates the NLRP3 inflammasome or reactive oxygen species generation in the vascular wall has not been directly investigated and remains an open question.

The remainder of the vascular-inflammation evidence currently derives from total AGP rather than from ORM2. Total AGP is an established inhibitor of platelet aggregation, and cleavage of AGP by the complement lectin-pathway serine protease MASP-1 has been proposed to counteract this platelet-inhibitory activity during the acute phase, since MASP-1 itself promotes platelet aggregation (34). Whether ORM2 specifically contributes to this platelet-modulatory activity, and whether ORM2 is a substrate for MASP-1 cleavage to the same extent as ORM1, has not been examined in published studies. Until isoform-resolved experimental data become available, these platelet-related observations should be regarded as AGP-level findings whose translational relevance to ORM2 in CAD remains presumptive rather than established.

### 4.4 ORM2 in acute coronary syndromes vs. chronic CAD

The temporal profile of total orosomucoid in acute coronary syndromes is that of a late-rising acute-phase reactant. In patients with MI, serum AGP concentrations peaked at days 4–7 after the index event, later than both CRP and haptoglobin, and this delayed rise distinguishes it kinetically from early-response markers such as CRP or troponin. In some series of unstable angina and non-Q-wave MI, orosomucoid elevation was independent of myocardial necrosis markers, raising the possibility that it reflects inflammatory activity rather than necrosis alone. In acute heart failure, which frequently co-occurs with CAD, circulating orosomucoid concentrations above a defined threshold were associated with increased risk of death or hospital readmission (30).

Importantly, every observation in this subsection derives from total orosomucoid or AGP rather than from ORM2-specific measurement. The prognostic value of ORM2 specifically, distinct from total orosomucoid, across the spectrum of stable CAD, unstable angina, and acute MI has not been evaluated in prospective cohort studies. This represents a defined gap that currently limits firm conclusions about ORM2 as a cardiovascular biomarker.

## 5 ORM2 as a molecular bridge: linking metabolic dysfunction to vascular inflammation

### 5.1 Shared inflammatory pathways in diabetes and CAD

T2DM and CAD share several underlying inflammatory mechanisms. In metabolic syndrome, chronic low-grade inflammation is characterized by elevated circulating concentrations of IL-1β, IL-6, and TNF-α, originating largely from chronically inflamed adipose tissue. These cytokines are associated with oxidative stress, which itself arises when reactive oxygen species (ROS) production exceeds antioxidant capacity (35). In adipose tissue from patients with metabolic syndrome, concentrations of CRP, IL-6, IL-1β, leptin, serum amyloid A, and MCP-1 are significantly elevated, while adiponectin concentrations are reduced (36).

The AGE-RAGE axis is another pathway shared by T2DM and CAD. In type 2 diabetic animal models, RAGE expression is upregulated in the aortic wall and is associated with increased NADPH oxidase activity and ROS generation (37). Hyperglycemia also contributes to increased production of AGEs, which are directly related to cellular and molecular dysfunction in the vascular wall (35). ORM2 is regulated by cytokines including IL-1β, TNF-α, and IL-6, placing it within these shared inflammatory signaling networks. Its hepatic expression is reduced in insulin-resistant and obese states, while the inflammatory pathways that regulate it are simultaneously activated in both T2DM and CAD (33).

### 5.2 ORM2 as an integrative mediator

ORM2 is proposed to connect hepatic metabolic function to systemic and vascular inflammation through several inter-organ signaling axes. The liver is the dominant site of ORM2 synthesis, and hepatic ORM2 expression is sensitive to cytokine-driven regulation and metabolic signals. In obese and insulin-resistant states, hepatic ORM2 concentrations are reduced (12). This reduction is associated with loss of AMPK-mediated suppression of hepatic *de novo* lipogenesis, which may contribute to increased lipid substrate availability relevant to atherosclerosis development (12).

ORM2 also mediates liver-to-adipose tissue communication. Hepatic ORM2 overexpression suppresses IFN-*γ*/STAT1 signaling in inguinal white adipose tissue, improving glucose homeostasis (11). This liver-adipose cross-talk is consistent with the broader concept of hepatokine-mediated inter-organ signaling. A liver-perivascular adipose tissue-blood vessel axis has been described for other hepatokines, where liver-secreted factors modulate perivascular adipocyte secretome and contribute to vascular inflammation and atherosclerosis (38). ORM2 may operate through a comparable mechanism. Administration of recombinant ORM2 reduced atherosclerosis in preclinical mouse models (12), and Orm2 deletion in mice results in exacerbation of steatohepatitis and increased hepatic fatty acid uptake via the Erk1/2-PPAR*γ*-Cd36 pathway (9). ORM2 also reprograms macrophages through IP3R2-dependent calcium signaling (13). Together, these observations support a model in which ORM2 may participate in the adipose–liver–vessel axis, with its deficiency in metabolic disease potentially amplifying both hepatic lipogenic and vascular inflammatory processes.

### 5.3 Interaction with other acute-phase proteins and adipokines

ORM2 belongs to a broader network of acute-phase proteins and adipokines that collectively regulate cardiometabolic risk. In patients with T2DM, serum AGP (orosomucoid) concentrations are positively correlated with CRP (r = 0.36, *P* < 0.05) and fibrinogen (r = 0.35, *P* < 0.05) (39). This co-elevation suggests that orosomucoid, CRP, and fibrinogen are co-regulated components of the same inflammatory response.

The adipokine profile in metabolic syndrome includes elevated leptin, reduced adiponectin, and dysregulated resistin and serum amyloid A (36, 40). These adipokines are associated with cardiovascular risk, and several biomarkers including fibrinogen, CRP, serum amyloid A, leptin, and resistin have been proposed as mediators linking obesity, insulin resistance, and cardiovascular disease. Adiponectin concentrations are inversely correlated with hsCRP and insulin resistance markers in metabolic syndrome (36).

### 5.4 The bidirectional behavior of ORM2: A critical synthesis

A central interpretive challenge in the ORM2 literature is the apparent contradiction between its protective and pathological associations. In preclinical mouse models, hepatic ORM2 expression is reduced under obesity and metabolic stress, and recombinant ORM2 administration ameliorates steatosis, steatohepatitis, and atherosclerosis (9, 10, 12). In human data from the KADEM cohort, however, circulating ORM2 is elevated, not reduced, in subjects with established hepatic steatosis and insulin resistance (30). Three complementary explanations resolve this paradox without requiring contradictory biology.

First, ORM2 has a dual identity as both a tonic hepatokine and an inducible acute-phase reactant. Hepatic intracellular ORM2 represents the protective pool, suppressing lipogenesis via IP3R2/AMPK/SREBP-1c signaling and improving insulin sensitivity through IFN-*γ*/STAT1 suppression, whereas circulating ORM2 in disease is driven upward by cytokine-mediated acute-phase induction involving IL-1β, IL-6, and TNF-α (11, 12, 26). These two pools are not the same biological signal and can move in opposite directions in the same individual. Second, animal and human studies likely capture different temporal stages of disease: an early protective hepatokine response that fails over time, followed by superimposition of an acute-phase inflammatory rise, a trajectory analogous to that described for other hepatokines and adipokines in cardiometabolic disease. Third, analogous to leptin, insulin, and FGF21 resistance, circulating ORM2 may rise without exerting a proportionate biological effect, either because target-cell signaling becomes desensitized or because inflammation-associated glycoforms differ in receptor engagement from the protective glycoforms of the physiological state (16, 27, 28).

ORM2 should therefore not be characterized as simply protective or simply harmful. Its biological output depends on source compartment, temporal phase of disease, and glycoform profile, and mechanistic statements about ORM2 should specify which of these dimensions is being referenced. Isoform-resolved and glycoform-resolved longitudinal assays will be required to formally test these explanations in human cardiometabolic disease.

### 5.5 Proposed conceptual framework

**Figure 1:**
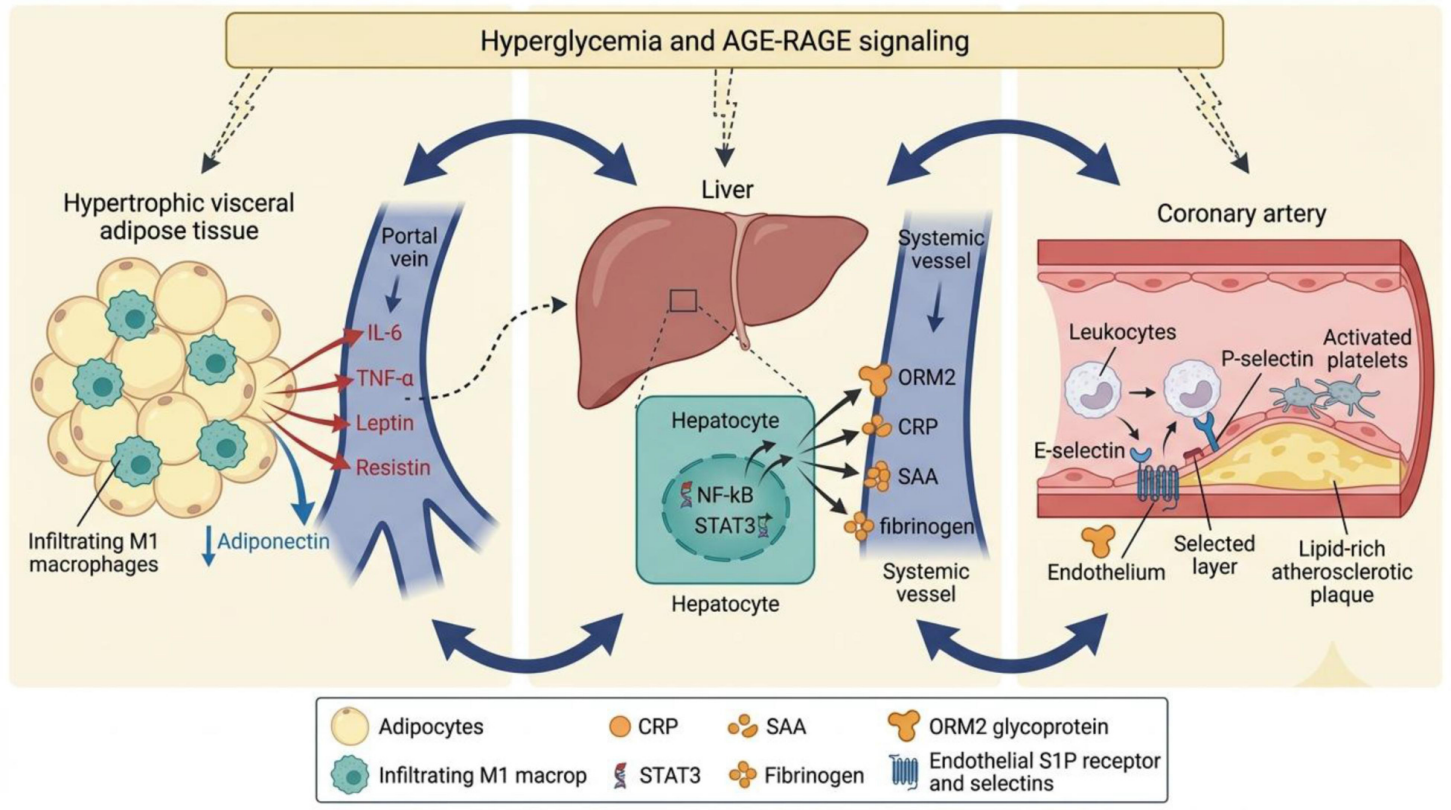
ORM2 as an integrative mediator in the adipose-liver-vessel axis. Visceral adipocytes release IL-6, TNF-α, leptin, and resistin into the portal vein, while adiponectin secretion is reduced. Portal cytokines activate hepatocyte NF-*κ*B and STAT3, driving acute-phase secretion of ORM2, CRP, SAA, and fibrinogen. In the coronary artery, circulating ORM2 is proposed to modulate endothelial selectins, leukocyte adhesion, and platelet activation; these vascular-compartment mechanisms remain hypothesis-generating. CRP, C-reactive protein; IL-6, interleukin-6; ORM2, orosomucoid 2; S1P, sphingosine-1-phosphate; SAA, serum amyloid A; TNF-α, tumor necrosis factor-alpha.

Figure 1 illustrates the proposed conceptual framework positioning ORM2 at the center of the adipose-liver-vessel axis in cardiometabolic disease. The figure depicts three interconnected compartments: (1) Adipose Tissue, showing expanded visceral adipocytes releasing IL-6, TNF-alpha, leptin, and resistin into the portal circulation, with reduced adiponectin secretion indicated by a downward arrow; (2) Liver, where portal-derived cytokines activate hepatocyte NF-kB and JAK-STAT3 signaling, leading to upregulated transcription and secretion of ORM2 alongside CRP, SAA, and fibrinogen into the systemic circulation; and (3) Coronary Vasculature, where circulating ORM2, through glycosylation-dependent interactions with endothelial selectins and sphingosine-1-phosphate receptors, may influence leukocyte adhesion, vascular permeability, and platelet reactivity; however, these vascular-specific mechanisms have not been directly investigated (1, 11). The proposed mechanistic framework places ORM2 as a central candidate mediator within a tri-compartment adipose–liver–coronary vasculature axis is summarized in Figure 1, which integrates adipose derived cytokine drive, hepatic acute phase amplification and endothelial and platelet responses into a single model of metabolic vascular coupling.

Importantly, direct experimental evidence demonstrating ORM2-mediated effects within the vascular wall remains limited, and these proposed mechanisms should be interpreted as hypothesis-generating.

## 6 ORM2 as a clinical biomarker and therapeutic target

### 6.1 Biomarker potential

Most of the available biomarker literature for the orosomucoid system in cardiometabolic disease derives from measurements of total orosomucoid or AGP rather than ORM2-specific assays, and this distinction is critical when interpreting translational relevance.

In diabetic kidney disease, total urinary orosomucoid excretion rate is independently associated with diabetic nephropathy, with an odds ratio of 3.10 (*P* < 0.0001), and increases progressively across normo-, micro-, and macroalbuminuria stages (32). In the prospective Malmö cohort, total orosomucoid was one of five inflammation-sensitive plasma proteins associated with myocardial infarction incidence over an 18-year follow-up (41). These findings indicate that circulating orosomucoid carries prognostic information in cardiometabolic disease but do not, on their own, establish ORM2-specific biomarker performance, since conventional immunoassays do not distinguish ORM1 from ORM2.

**Figure 2:**
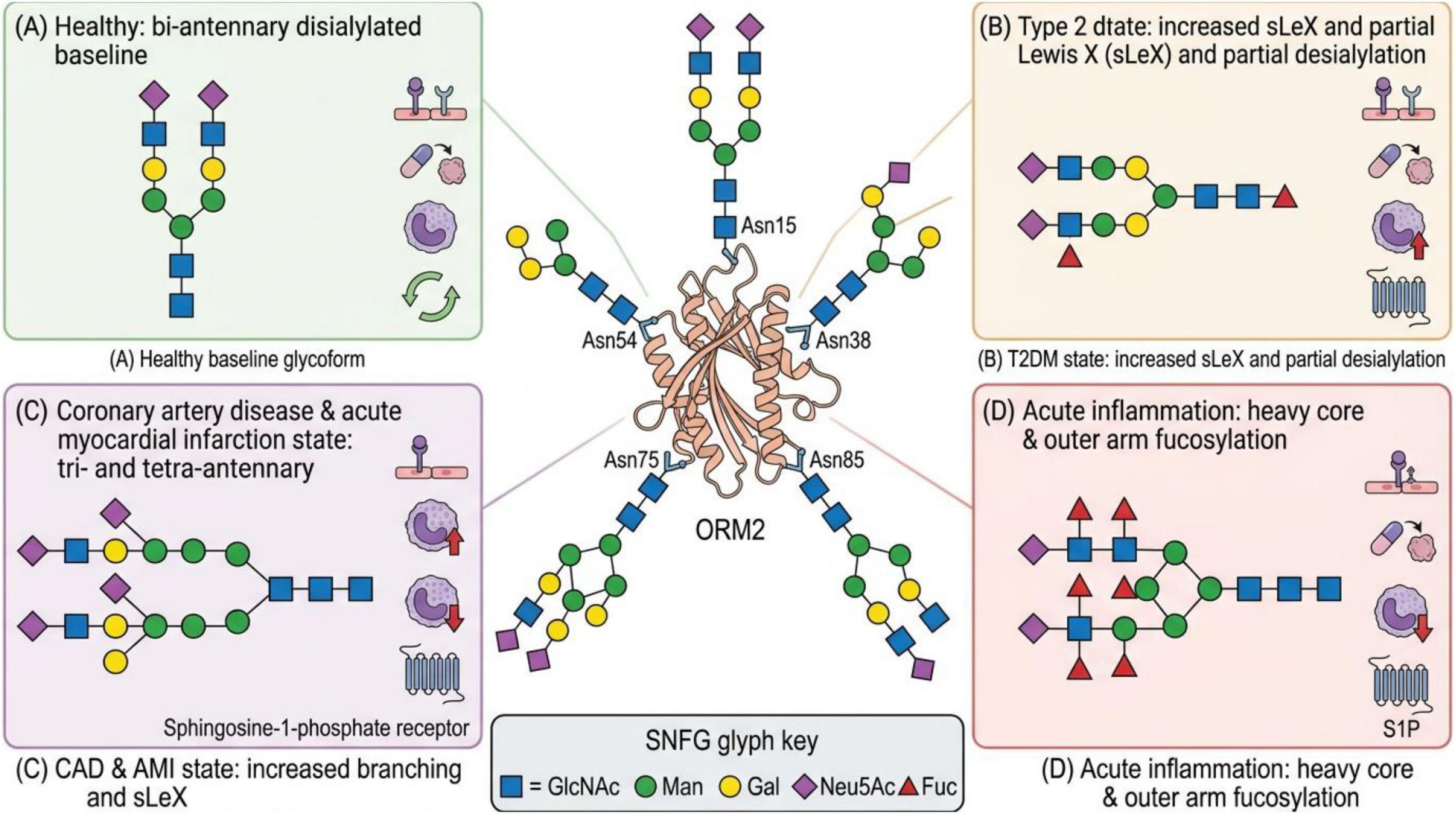
Disease-specific glycoform heterogeneity of ORM2 and its functional consequences. ORM2 carries five N-linked glycans at Asn15, 38, 54, 75, and 85. **(A)** Healthy baseline shows bi-antennary disialylated glycoforms. **(B)** T2DM shifts toward increased sialyl Lewis X (sLeX) and partial desialylation. **(C)** CAD and acute MI show tri- and tetra-antennary branching with sLeX. **(D)** Acute inflammation features heavy core and outer-arm fucosylation. Glycoform–disease associations derive largely from total AGP data and are hypothesis-generating for ORM2. AGP, alpha-1-acid glycoprotein; CAD, coronary artery disease; MI, myocardial infarction; ORM2, orosomucoid 2; sLeX, sialyl Lewis X; T2DM, type 2 diabetes mellitus.

Direct human ORM2-specific biomarker data have recently emerged from the KADEM study (30), in which plasma ORM2 was measured by ELISA in 449 Kuwaiti adults. In this cohort, ORM2 was an independent predictor of hepatic steatosis severity (adjusted OR = 1.005; 95% CI: 1.002–1.007), and a composite ROC model incorporating ORM2 alongside metabolic variables achieved an AUC of 0.864 for metabolic dysfunction-associated fatty liver disease. The KADEM data (57) therefore represent the strongest currently available human ORM2-specific biomarker signal, although prospective validation in independent cohorts and in CAD-specific populations has not yet been performed. The glycosylation state of AGP changes in inflammatory and disease conditions, and N-glycan structural variation in AGP, including differences in branching and sialylation, alters drug-binding affinity and protein function (27, 28). Desialylated AGP also shows measurably different ligand binding compared with native forms (16). ORM2 glycoform profiling has therefore been proposed as a potential precision approach for disease staging and biomarker refinement in cardiometabolic disease. However, almost all clinical glycoform data currently available derive from total AGP rather than from isolated ORM2, and no standardized, clinically validated ORM2-specific glycoprofiling assay is currently available. Translation of glycoform analysis into routine clinical use will therefore require dedicated assay development, inter-laboratory standardization, and prospective validation in defined patient cohorts before it can inform clinical decision-making. Table 2 lists the disease-specific glycoform fingerprints of ORM/AGP reported across cardiometabolic, inflammatory, and oncological conditions, and is provided as a research-stage reference framework rather than as a clinically implementable assay panel. The molecular logic by which a single ORM2 polypeptide may convey disease-specific information through its N-glycan repertoire is illustrated in Figure 2, which maps proposed glycoform transitions (fucosylation, sialylation, and antennary branching) to candidate functional consequences in diabetes and CAD; these mappings should be interpreted as hypotheses derived largely from total AGP data and are not yet validated for ORM2 specifically.

**Table 2:** Disease-specific ORM/AGP glycoform fingerprints.

Disease/condition	Dominant AGP glycoform change	Functional/clinical consequence	References
Hepatocellular carcinoma	Increased core and outer arm fucosylation; rise in multifucosylated tetra-antennary glycans; falling bi-antennary fraction	Serum fucosylated AGP outperforms AFP in early HCC detection and differentiates HCC from cirrhosis across HBV, HCV and alcoholic etiologies	(43, 44)
Chronic hepatitis/cirrhosis	Shift towards more branched glycans and increased sialyl Lewis X epitopes; reduced total AGP in end stage liver disease	Tracks fibrosis progression; serum AGP concentration falls as hepatic synthetic reserve declines, limiting its utility as a pure inflammatory marker in cirrhosis	(45, 46)
Rheumatoid arthritis	Decreased bi-antennary and increased tri- and tetra-antennary branching; increased sialyl Lewis X expression	Branching index correlates with disease activity score and reflects ongoing leukocyte activation and joint inflammation	(14, 47)
Pregnancy	Predominance of bi-antennary mono-sialo glycans; reduced branching compared with non-pregnant controls	Immuno-tolerogenic glycoforms may support fetal tolerance; distinct from inflammation-driven profiles and useful for interpreting AGP during gestation	(14, 48)
Ovarian and other gynecological cancers	Increased sialyl Lewis X branching and aberrant tri-/tetra-antennary glycans on AGP detected by CZE and MRM proteomics	Glyco-profile-based scores complement CA-125 for discriminating malignant from benign pelvic masses	(49, 50)
Coronary artery disease/acute myocardial infarction	Elevated total AGP with increased branching and sialyl Lewis X; isoform shift on capillary electrophoresis during acute events	AGP is among the inflammation-sensitive plasma proteins whose elevation predicts fatal coronary events and post-MI mortality at 7-year follow up	(51, 52)
Heart failure (acute and chronic)	Raised AGP concentration with branched glycan enrichment; pattern distinct from nutritional hypo-albuminemia	AGP is an independent predictor of post-discharge mortality and rehospitalization; combination with omentin (OROME score) refines risk stratification	(30, 53, 54)

AFP, alpha-fetoprotein; AGP, alpha-1-acid glycoprotein; CA-125, cancer antigen 125; CZE, capillary zone electrophoresis; HBV, hepatitis B virus; HCC, hepatocellular carcinoma; HCV, hepatitis C virus; IBD, inflammatory bowel disease; MI, myocardial infarction; MRM, multiple reaction monitoring; ORM, orosomucoid; OROME, orosomucoid + omentin score; T2D/T2DM, type 2 diabetes mellitus; UC, ulcerative colitis.

Proteomics-based risk stratification models that incorporate inflammatory and metabolic proteins have shown improved prediction of atherosclerotic cardiovascular disease events beyond conventional scores; whether ORM2 contributes independently to such multi-marker models warrants prospective evaluation (42).

In summary, the current ORM2 biomarker evidence base supports further investigation but does not yet meet the criteria for clinical translation. Large prospective cohort studies measuring ORM2 separately from ORM1, using standardized ORM2-specific assays and ideally complemented by glycoform analysis, are required before ORM2 can be deployed as a clinical biomarker in T2DM or CAD.

### 6.2 Therapeutic targeting of ORM2

The current evidence supporting ORM2 as a therapeutic target derives entirely from preclinical animal studies, and this should be stated explicitly when discussing clinical applicability. Intraperitoneal administration of recombinant ORM2 protein improved liver steatosis, steatohepatitis, and atherosclerosis in mouse models without adverse effects on body weight or food intake (12).

Pharmacological administration of recombinant ORM2 also ameliorated hepatic steatosis, inflammation, and fibrosis through the Erk1/2/PPAR*γ*/CD36 pathway (9). In an insulin-resistant polycystic ovary syndrome model, supplementation with recombinant ORM2 improved ovarian morphology and white adipose tissue UCP1 expression (10). These studies establish a proof-of-concept for recombinant ORM2 administration in metabolic disease but do not, in isolation, support immediate clinical translation. Several critical translational steps have not yet been undertaken. No published human Phase I or Phase II clinical trials of recombinant ORM2 have been reported. Pharmacokinetic profiling in humans, including half-life, tissue distribution, and the influence of glycoform composition on clearance, has not been characterized. Immunogenicity, particularly for ORM2-Fc fusion proteins, has not been assessed in human subjects. Safety and dosing data are not available, and the long-term consequences of pharmacologically elevating circulating ORM2, including potential effects on drug-binding capacity for co-administered medications, have not been studied. Clinical development of recombinant ORM2 therapy will therefore require dedicated translational programs encompassing manufacturing, regulatory, pharmacokinetic, and safety studies before efficacy trials in T2DM or CAD populations can be initiated. In parallel, currently approved cardiometabolic drugs that modulate inflammatory signaling provide context for the potential therapeutic positioning of ORM2-directed approaches. SGLT2 inhibitors modulate inflammatory signaling pathways in a glucose-independent manner (33), are proposed to indirectly target the IL-1β pathway and reduce low-grade inflammation in patients with T2DM at high cardiovascular risk (55), and empagliflozin has been shown to improve endothelial dysfunction through redox-dependent mechanisms (56).

These agents illustrate that anti-inflammatory mechanisms within cardiometabolic pharmacotherapy are clinically tractable, but they do not constitute evidence for ORM2-targeted therapy and are presented here only as a parallel reference framework.

### 6.3 Precision medicine implications

Glycoform-dependent variation in ORM2/AGP function (16, 27) suggests that ORM2 glycosylation profiling could in principle have pharmacogenomic relevance, since patients with distinct glycoform distributions may differ in ORM2-mediated drug transport and inflammatory modulation. This precision medicine perspective should be regarded as a research-stage hypothesis rather than as an established clinical strategy, since no human studies have demonstrated that ORM2 glycoform profiling alters clinical decision-making or improves patient outcomes. Mendelian randomization analysis has been used to assess causal relationships between circulating proteins and cardiovascular endpoints, including in the pathway between metabolic liver disease and atherosclerotic cardiovascular events (42).

**Figure 3:**
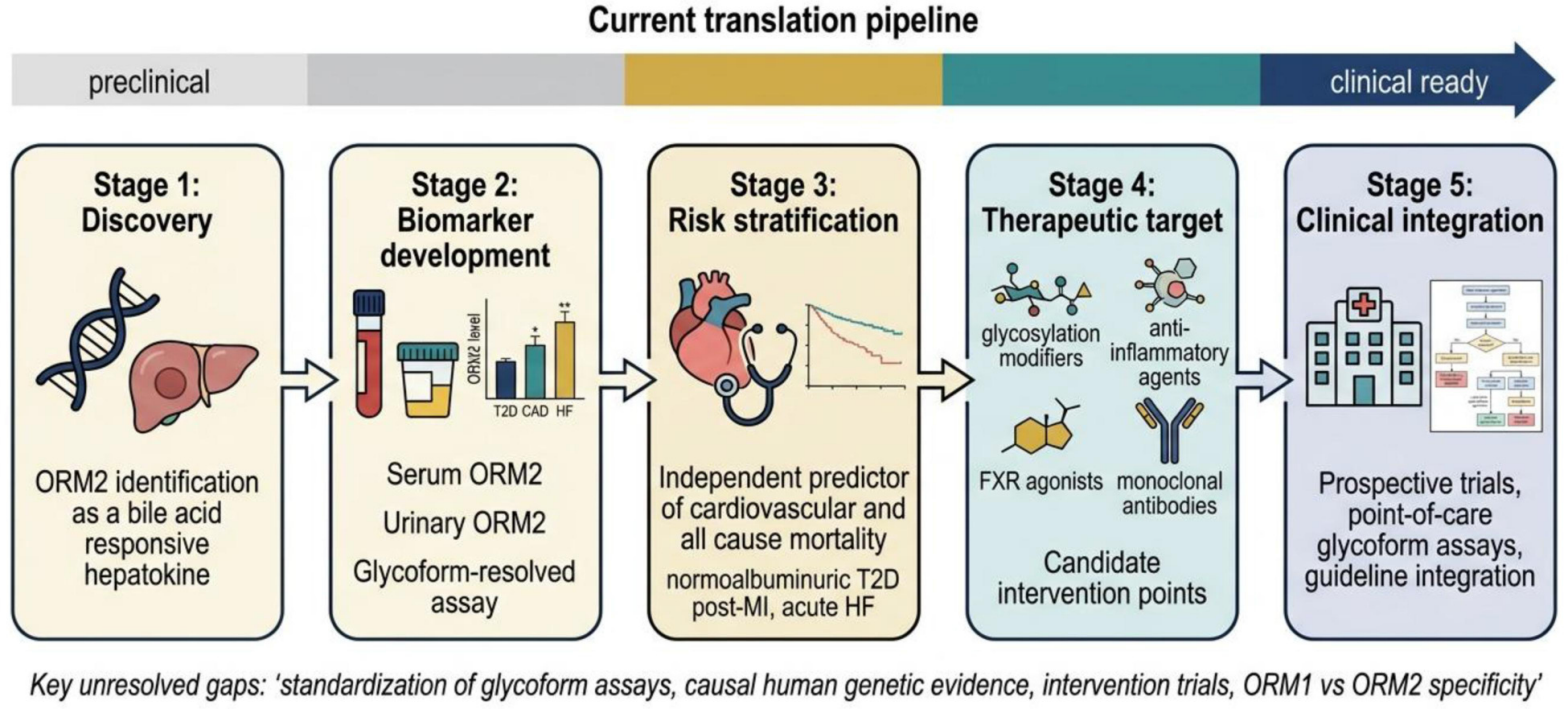
Clinical translation roadmap for ORM2 in cardiometabolic medicine. A staged framework from preclinical discovery to clinical integration. Stage 1: ORM2 identification as a bile acid–responsive hepatokine. Stage 2: development of serum, urinary, and glycoform-resolved ORM2 assays. Stage 3: risk-stratification in normoalbuminuric T2DM, post-MI, and acute HF cohorts. Stage 4: candidate therapeutic interventions targeting ORM2 pathways. Stage 5: prospective trials and guideline integration. Key unresolved gaps remain in assay standardization, causal genetic evidence, and ORM1/ORM2 specificity. HF, heart failure; MI, myocardial infarction; ORM2, orosomucoid 2; T2DM, type 2 diabetes mellitus.

Applying such genetic-instrument approaches to ORM2 specifically would provide one of the most informative near-term pathways for clarifying its causal role in T2DM and CAD. A translational roadmap that positions ORM2 within the diagnostic, prognostic, and therapeutic landscape of cardiometabolic medicine is presented in Figure 3, which summarizes the current evidence tier for each potential clinical application and highlights the pre-analytical, assay, and trial gaps that must be closed before ORM2-based tools can enter routine clinical use. The figure is intended as a research-stage roadmap rather than as a clinical implementation pathway.

## 7 Current gaps, limitations, and future directions

### 7.1 Methodological limitations in existing studies

Several methodological limitations affect the existing evidence on ORM2. Most clinical studies have measured total orosomucoid or total AGP rather than ORM2 specifically, making it difficult to attribute findings to ORM2 as distinct from ORM1. The two proteins are co-expressed and share structural features, but their regulatory and functional profiles differ. Standardized assays specific to ORM2 protein are not widely available. Existing AGP assays typically measure the aggregate of both isoforms. Similarly, methods for quantifying ORM2 glycoforms in clinical samples are not standardized. Most animal studies used either Orm2 knockout or recombinant ORM2 overexpression models without tissue-conditional manipulation, which limits interpretation of organ-specific contributions. Cross-sectional designs in clinical studies preclude assessment of temporality or causal direction. Several fundamental questions about ORM2 in cardiometabolic disease are unresolved. Whether changes in ORM2 expression are causally related to diabetes or CAD pathophysiology, or are secondary to the inflammatory state, is not established. Mendelian randomization studies using genetic instruments for ORM2 expression would provide a method to assess causality, as has been applied for other proteins in the pathway between metabolic liver disease and atherosclerotic cardiovascular outcomes. The respective contributions of ORM1 and ORM2 to the clinical observations attributed to total orosomucoid are not known. Sex-based differences in ORM2 expression or function have not been systematically evaluated in cardiometabolic contexts. The role of ORM2 in other cardiometabolic conditions, including heart failure and peripheral artery disease, is unexplored. Direct clinical studies measuring ORM2 specifically in T2DM and CAD cohorts, with comparison to ORM1 and established inflammatory markers, are not available in the published literature.

### 7.2 Recommended future research directions

Based on the gaps identified, the following research directions are proposed. First, large-scale prospective cohort studies that measure ORM2-specific concentrations, ideally alongside ORM1 and established biomarkers, would permit assessment of ORM2 as an independent predictor of cardiovascular events in patients with T2DM. Serial measurements across disease stages would be required to characterize ORM2 dynamics. Second, Mendelian randomization using ORM2 expression quantitative trait loci as genetic instruments would allow causal inference regarding ORM2 in T2DM and CAD outcomes. Third, tissue-specific conditional knockout models of Orm2, targeting the liver, adipose tissue, and myeloid cells separately, would permit organ-specific functional characterization beyond what global knockouts allow. Fourth, multi-omics integration encompassing proteomics, glycomics, and metabolomics would map the ORM2 interactome in disease states. Proteomics-based approaches have demonstrated the capacity to improve risk stratification for atherosclerotic cardiovascular events in metabolic liver and similar approaches applied to ORM2 glycoforms could refine biomarker performance. Fifth, standardized ORM2 glycoprofiling assays are needed before glycoform analysis can be translated into clinical practice, as current glycomic analyses of AGP are not standardized across laboratories.

## 8 Conclusion

ORM2 is a hepatocyte-derived acute-phase protein with documented roles in hepatic lipid metabolism, insulin sensitivity, and macrophage regulation. Its expression is reduced in obesity and insulin-resistant states, and circulating orosomucoid concentrations are associated with diabetic nephropathy, microalbuminuria, and myocardial infarction risk in clinical data. At the mechanistic level, ORM2 operates at the intersection of hepatic acute-phase signaling, adipose tissue inflammation, and vascular biology, pathways that are co-activated in both T2DM and CAD.

Preclinical administration of recombinant ORM2 reduced atherosclerosis, hepatic steatosis, and steatohepatitis in mouse models, providing an early experimental basis for therapeutic translation. ORM2 and its glycoforms may also serve as precision biomarkers for cardiometabolic risk stratification, though dedicated prospective studies using ORM2-specific assays are needed to establish clinical utility.

Whether ORM2 has a causal role in cardiometabolic disease pathophysiology, what distinguishes its function from ORM1, and how sex and ethnicity influence its expression remain unanswered. These questions require investigation through prospective cohort studies, Mendelian randomization analyses, and tissue-conditional experimental models. ORM2 warrants systematic evaluation as both a molecular mediator and a candidate clinical target in the shared pathophysiology of diabetes and coronary artery disease.
